# Post-splenectomy response in adult patients with immune thrombocytopenic purpura

**DOI:** 10.4103/0973-6247.45255

**Published:** 2009-01

**Authors:** Avinash Supe, Maulik Parikh, Ramkrishna Prabhu, Chetan Kantharia, Jijina Farah

**Affiliations:** *Department of Surgical Gastroenterology, Seth G S Medical College and K E M Hospital, Parel, Mumbai - 400 012, India*; 1 *Department of Haematology, Seth G S Medical College and K E M Hospital, Parel, Mumbai - 400 012, India*

**Keywords:** Idiopathic purpura, surgical therapy

## Abstract

Splenectomy has been the conventional surgical treatment for patients with Immune Thrombocytopenic Purpura (ITP).

**Aim:**

To define response to surgical therapy, pre operative factors influencing outcome and tolerability of surgery in adult patients undergoing splenectomy for ITP.

**Method:**

We analyzed prospectively maintained data of 33 patients who were diagnosed as ITP and underwent splenectomy over the last 10 years. The age, presenting complaints, bleeding manifestations, clinical details and other investigations were noted. Details of immediate pre-operative administration of blood transfusions, platelet transfusions and other forms of therapy were also recorded. Operative details with regards to blood loss and the presence of accessory spleens were obtained. Postoperative course in terms of clinical improvement, rates of complications and platelet counts was also noted.

**Results:**

Skin petechiae and menorhhagia were common presenting symptoms in patients (mean age 26.5±10.5 yrs) with ITP. Eighteen patients underwent splenectomy for failure of therapy and fifteen for relapse on medical treatment. Mean platelet bags transfused in immediate pre-operative period were 2.8±0.8. Mean intra-operative blood loss was 205±70.5 ml. Accessory spleens were removed in 1 case (3.03%). The immediate postoperative response was complete in 19 cases (57.58%) and partial in 13 cases (39.39%). The platelet counts increased significantly from 23142±12680/ µL (Microliter) (mean ± SD) preoperatively to 170000±66000/µL (Microliter) within 24-48 hours after splenectomy (*P* < 0.05). The mean platelet count was 165000+66000/µL (Microliter) at the end of one month when steroids were tapered off gradually. Four patients (12.12%) had complications (one each of wound hematoma, wound infection, splenic fossa collection and upper GI hemorrhage) in postoperative period but all responded to therapy. One relapsed patient was detected with accessory spleen and responded after re-surgery. Response to splenectomy was better in young patients and in those patients who had higher immediate post-splenectomy thrombocytosis.

**Conclusions:**

Splenectomy is safe and effective therapy in ITP patients with no response to steroids and relapse after medical therapy. Response to splenectomy was more in young patients and in those patients who had higher immediate post-splenectomy thrombocytosis.

## Introduction

Since Kaznelson performed the first splenectomy for Immune Thrombocytopenic Purpura (ITP) in 1916, it was considered to be the treatment of choice[1–5] for ITP, before the introduction of glucocorticoids more than 50 years ago. For the past 50 years, splenectomy has remained a conventional treatment for adults with ITP who do not respond to glucocorticoids or who are steroid dependant. Even in the present era, the response rates of splenectomy are 50-85% compared to the response rates of 15-30% when steroids are administered.[5–7] Complete and partial responses in patients treated with steroids is about 65-85%. Since sustained responses after discontinuation of the drug occur in only 25% or less, surgical therapy is preferred as it usually gives complete response. However there is no Indian data on response to splenectomy in terms of maintenance of safe platelet counts, preoperative predictability and surgical outcomes.

The purpose of this study was to evaluate response to surgical therapy and determine factors influencing outcome in adult patients undergoing splenectomy for ITP.

## Materials and Methods

Eighty-five patients underwent splenectomy for various hematological disorders in the period between 1996 and 2006 in KEM Hospital. The study includes prospectively maintained data of the 33 patients who underwent surgery for ITP. ITP was diagnosed with presence of a platelet count less than 100,000 /µL (Microliter) in the absence of any other secondary cause with normal or increased megakaryocytes. Bone marrow IgG against platelets was assessed in only a few patients. The reason why the splenectomy was carried out in each condition was noted.

The age of the patients, the primary presenting complaints, bleeding manifestations, presence of a palpable spleen, duration of steroid therapy and other clinical details were obtained. The pre-operative administration of blood transfusions, platelet transfusions and other forms of therapy were noted. Operative details with regards to presence of accessory spleens, operative blood loss as well as postoperative complications were noted.

Postoperative response was defined as complete (CR), partial (PR) and no response (NR) - depending on platelet counts at the end of one month. (CR- platelet count > 100,000 / µL (Microliter), PR- 50,000 to 100,000 / µL (Microliter), NR- counts < 50,000/ µL (Microliter)) These counts were obtained immediately after splenectomy and at the end of one month after surgery.

The statistical analysis was carried out by using Student's “t” test.

## Results

The predominant presenting features in our series were skin bleeding and menorrhagia Table 1. On examination, 10 patients had petechiae and 4 patients had ecchymotic patches. The mean age of patients (M 11, F 22) was 26.52 yr. ± 10.5. None of the patients had a palpable spleen. The hemoglobin of the patients varied from 6.4 g/dl to 12.2 g/dl- the mean being 10.2 ± 1.3 (mean ± S.D.). The preoperative platelet count varied from 7000 to 60,000/µL (Microliter) (mean±1SD 23,142 ± 12,680.3). The coagulation profile (PT, PTTK) was normal in all the patients. Bone marrow aspiration was done in 15 of the patients and was diagnostic. The other cases were diagnosed on clinical features and platelet counts.

**Table 1:** Presenting clinical symptoms in patients with ITP (n=33)

Clinical features	No. of patients
Skin bleeding alone	9
Menorrhagia alone	5
Mucosal bleeding alone (epistaxis / bleeding gums)	4
Skin bleed + Menorrhagia	4
Skin bleed + Menorrhagia + Mucosal bleeding	2
Menorrhagia + Mucosal bleeding	1
Skin bleeding + Mucosal bleeding	4
Skin bleeding + GI bleeding	2
Wound hematoma	1
Subdural hematoma	1

All the patients had received steroids (prednisone) prior to surgery. Methyl prednisone was administered to 4 patients as an emergency measure and was eventually converted to prednisone. Seven patients had received platelet transfusions for extremely low values. One patient received immunoglobulin while undergoing hysterectomy. Eighteen patients underwent splenectomy for failure to respond to steroids and the other 15 patients had relapse after medical therapy. Relapse was defined as the appearance of bleeding manifestations or the fall in platelet counts in patients in whom the steroids were being tapered or had been stopped. Six patients had a fall in platelet counts, 4 patients developed ecchymotic patches and 5 patients developed serious bleeding (malaena in two, subdural hematoma in one and menorrhagia in two). The time interval between the first presentation and the splenectomy was 3.2 ± 1.9 months in case of the non-responders. It was 6.9 ± 3.1 months in case of the relapsers. The mean duration in patients who experienced a complete response was 4.2 ± 2.9 months, while in those patients who had a partial or no response it was 6.0 ± 2.7 months (*P* > 0.05). In our series there was no significant difference in response between the steroid responders and non-responders (*P*>0.05).

As platelets are destroyed within an hour post transfusion by spleen, all the patients received intra-operative platelet transfusion during surgery after clamping of the splenic artery. Mean platelet bags administered to the patients were 2.80 ± 0.80. Five patients received a fourth bag because of diffuse oozing from the splenic bed, which was controlled by platelet transfusion and compression. Eighteen patients were operated through a midline incision while 13 through a left sub costal incision. Two patients were operated through laparoscopy. Six patients (28.6%) had adhesions. The operative time was 99±14 min and average blood loss was 205±71 ml. The average weight of the spleen was 270±21 gm. A single accessory spleen was present in the greater omentum of one of the patients.

Complications were seen in four patients (12.12%). One patient had wound infection. One patient developed a wound hematoma, which had to be evacuated. One patient developed a splenic fossa collection, which was tapped under ultrasound guidance. Another patient had coffee-ground nasogastric aspirate on the first postoperative day, which stopped afterwards.

Platelet counts 24 hours after surgery varied from 35,000 to 3,75,000 / µL (Microliter) (Mean ±1SD 1, 70,000 ± 66,000) (Pre vs. Post - *P*<0.05). At 1 month after surgery, the counts varied from 30,000 to 2,90,000/µL (Microliter) (Mean± 1SD 1,65,000 ± 66,000) (24 hours vs. one month *P*>0.05). Thirty patients continued steroids postoperatively and the steroids were then tapered and gradually stopped over 1 to 3 months. There was not much change in platelet counts at the end of three months. Three patients did not receive any steroids post-operatively.

Complete Response (CR) was obtained in 19 patients (57.58%), Partial Response in 13 patients (39.39 %) and one patient failed to respond to surgery. This patient received Azathioprine and had to be given platelets for a large wound hematoma. Complete Response (CR) occurred more in patients below 30 years of age (*P* < 0.005). The patients with complete response had less severe manifestations than the patients with partial or no response (*P* < 0.05). One patient was symptomatic after two years and was detected to have an accessory spleen. He was re-operated and had complete response.

Preoperative factors such as age were also at work, where complete response was more in younger patients. CR was also seen in those patients, who had higher initial post-operative platelet counts (*P* < 0.05).

## Discussion

Immune thrombocytopenic purpura is an autoimmune condition characterized by the binding of antiplatelet antibodies to platelets followed by phagocytosis by the reticuloendothelial system, mostly in the spleen. The spleen also serves as the site of production of the antibodies.[3] Steroids are considered to be the first line of therapy and splenectomy is offered only in symptomatic patients who fail to respond to steroids or those who relapse on or after therapy.[4,5] Sustained response to steroids has been reported to be between 15-30%.[6–8] Steroids may act by (a) decreasing consumption of antibody coated platelets by the spleen or bone marrow, (b) reduce antibody production by spleen and bone marrow and (c) increase marrow platelet production by undetermined mechanisms. Splenectomy removes the major site of platelet destruction and improves the platelet survival times.[9] It also reduces antibody production.

Splenectomy was technically more demanding in six cases in our series, where there were adhesions. Accessory spleen was noted in one patient in our series (3.03%), which has been reported by others.[10] The recurrence of hematological diseases has been found to occur following surgery after variable periods. It has been known to occur in patients with ITP from 7 months to several years later.[10,11] Because of the high incidence of accessory spleens, the risk of recurrence of the disorder, and the difficulty experienced in locating them and excising them during repeat surgery, serious attempts must be made to look for them during the primary splenectomy itself. Though not used by us, localization has been attempted by the use of various radionuclide scans. Indium 111 labeled platelet scan has proved to be the most sensitive.[12,13] We have given only 2-4 units of platelets per adult patient as compared to the recommended 4-6 units. This is due to financial and supply constraints. However we have not noticed any morbidity or excessive bleeding. As platelets are destroyed within one hour after transfusion, we ensured that platelets were given only after splenic artery ligation. This may have reduced the units of platelets transfused.

The incidence of postoperative complications in this series is 12.12%. Splenic fossa collection occurred in one case and was successfully tapped under ultrasonographic guidance. Increased incidence of postoperative collections has been reported in patients where the splenic fossa has been drained,[14] We do not prefer keeping drains in splenic fossa. There were 2 cases of wound related morbidity in the series (one wound hematoma and one wound infection).

Over the years attempts have been made to determine factors, which would be prognostic of a satisfactory response to splenectomy. Age has been the most commonly studied factor with remission being found in the younger age groups as was confirmed in present series,[4,7,15] however others have not been able to confirm this.[3,8] There are conflicting reports regarding previous response to steroids as one of the factors for predicting response.[4,12,16,17] In our series there was no difference in response amongst responders and non- responders. In the present series the patients who had a complete response had higher post splenectomy platelet counts (*P* < 0.005). Fabris *et al*.[7] had also believed that immediate post splenectomy thrombocytosis could be an indicator of long term remission. In our series, we have also tried to determine whether the severity of the hemorrhagic manifestation could be a prognostic factor. A similar attempt to classify the bleeding and to study its predictiveness was made by Julia *et al*.[4] and they found that patients with skin and mucosal bleeding did worse than patients with mucosal bleeding alone who did worse than the patients with skin bleeding alone. In the present series we also noticed that the patients with complete response had less severe manifestations than the others.

The period between the presentation of the patient and the performance of splenectomy has also been reported to be of predictive value. There have been reports where longer duration of medical therapy has resulted in a poorer response.[18] We have not been able to confirm this. Two other factors that are considered to be predictive but could not be studied in this series are the IgG levels against platelets[12,15] and the site of platelet sequestration. It has been shown that the patients in whom the predominant site of platelet sequestration is the spleen, respond well to surgery.[12]

The spleen has a major role in the immunoprotection of an individual. Its role in phagocytosis of blood borne antigens, synthesis of antibodies and opsonins such as tuftsin and properdin are now well established.[19] The emphasis in the present day is on splenic conservation procedures due to the supposedly high incidence of post-splenectomy sepsis (4.4% in children under 16 years and .9% for adults).[20] Amongst these procedures, there are reports of partial splenectomy in various disorders.[1,21,22] Though these series demonstrate satisfactory results, partial splenectomy for hematological disorders leaves a number of issues unresolved. It has not been determined as yet how much splenic tissue has to be conserved with the aim of preserving the spleen's immune function and at the same time optimally treating the disorder for which the surgery is being carried out. By animal experiments it has been shown that 25-50% of the spleen is required for the maintenance of immune function.[23] This would mean that a considerable size of splenic mass will have to be conserved. Accessory spleens of a size of even 13 gm[10] have been shown to lead to recurrence of ITP. So, it seems somewhat illogical to do partial splenectomy leaving a splenic mass of 25-50% behind (which can almost surely lead to recurrence). Another issue that has to be addressed is whether the remaining splenic tissue can grow in size under the persisting stimulus of the disorder. The exact growth rate has not been determined, but there have been instances where accessory spleens missed during the primary splenectomy have been shown to be of large sizes at the time of repeat splenectomy in patients with recurrent ITP.[10] Follow-up is absolutely essential and patients have to be told of the possibility of repeat surgery in case of recurrence of the disease. Repeat surgery would be fraught with technical difficulties in view of the presence of postoperative adhesions. It is probable that the incidence of postsplenectomy sepsis has been overestimated especially in adults and that the actual incidence is minimal.[20] Thus it is essential to individualize the indication for partial splenectomy and weigh the benefits of the procedure against the drawbacks of recurrence.[4]

Like all other laparoscopic procedures, laparoscopic splenectomy appears to be an attractive alternative. Patients who undergo laparoscopic splenectomy have a shorter postoperative recovery and have reduced wound related morbidity.[24] The only drawback with laparoscopic splenectomy is the longer operative time[25] and the likelihood of missing accessory spleens. In our series, we had laparoscopic splenectomy in two patients.

In conclusion, splenectomy was safe and useful in steroid failures and relapsers in our series. The complete response rate in patients undergoing splenectomy in ITP in our series is 57.58% while partial response was seen in 39.39% of the patients. Age and post-splenectomy thrombocytosis may aid in predicting the response.
